# Hornets and Honey Bees: A Coevolutionary Arms Race between Ancient Adaptations and New Invasive Threats

**DOI:** 10.3390/insects12111037

**Published:** 2021-11-18

**Authors:** Federico Cappa, Alessandro Cini, Laura Bortolotti, Juliette Poidatz, Rita Cervo

**Affiliations:** 1 Dipartimento di Biologia, Università di Firenze, Via Madonna del Piano 6, Sesto Fiorentino, 50019 Firenze, Italy; cini.ales@gmail.com (A.C.); rita.cervo@unifi.it (R.C.); 2 Centre for Biodiversity and Environment Research, Department of Genetics, Evolution and Environment, University College London, Gower Street, London WC1E 6BT, UK; 3 CREA Research Centre for Agriculture and Environment, Via di Corticella n. 133, 40128 Bologna, Italy; laura.bortolotti@crea.gov.it; 4 Environment and Sustainability Institute, Penryn Campus, University of Exeter, Penryn TR10 9FE, UK; J.Poidatz@exeter.ac.uk

**Keywords:** *Vespa*, *Apis*, coevolution, Asian hornets, alien invasive species

## Abstract

**Simple Summary:**

Hornets of the genus *Vespa* and honey bees are the main characters of a coevolutionary arms race that is made evident by the conspicuous number of reciprocal adaptations evolved by both predator and prey. Through this ancient process predatory hornets and honey bees seem to have reached a fragile balance, which is lost when alien hornet species are accidentally introduced into new geographic areas. This is the case of Asian hornet species, such as *Vespa velutina* and *Vespa mandarinia*, which have invaded Europe and North America in recent decades, with a heavy impact on Western honey bees and commercial beekeeping. Here, we provide a comprehensive review of the existing studies that have investigated the interactions between hornets and honey bees throughout the world, highlighting the complexity of the specialized strategies adopted by Asian honey bees and their sympatric predators and the relative lack of coevolution and effective defenses of Western honey bees against invasive hornets.

**Abstract:**

Hornets and honey bees have a long history of coevolution resulting in a plethora of captivating adaptations and counteradaptations between predator and prey. From simple physiological mechanisms to complex behavioral strategies, some *Vespa* hornets have specialized in hunting honey bees, while the latter have put in place effective defenses to counteract their attack. Both hornets and honey bees have evolved the ability to detect the odors and the pheromones emitted by the other to locate the prey or to spot foraging predators. Hornets often rely on their bigger size, heavily armored body and destructive attacks, while honey bees differentiated collective defense responses finely coordinated to deter or kill the hornet menace. However, when new species of hornets and honey bees come into contact, the absence of coevolution can have a heavy impact on the defenseless bees. The evolutionary arms race between hornets and honey bees provides not only compelling examples of adaptations and counteradaptations between predator and prey, but could also represent a starting point for the development of effective and sustainable strategies to protect honey bees and beekeeping activities and to control invasive alien species of hornets.

## 1. Introduction

Among the arthropods that can be identified as natural enemies of honey bees (*Apis* spp., Hymenoptera, Apidae), a most fascinating and sometimes complex relationship regards the prey–predator evolutionary race between bees and hornets of the genus *Vespa* (Hymenoptera, Vespidae) [1,2,3,4,5,6,7]. This genus of predatory social hymenopterans represents one of the four genera in the subfamily Vespinae and it consists of 22 extant species [8,9,10]. Most of these species have an Asian distribution, with the highest diversity in terms of taxa in northern Indo-Malaya [3,9,11]. Only two species, *Vespa crabro* and *V. orientalis*, are naturally distributed outside of Asia, across Europe, around the Black Sea and the Caspian Sea (*V. crabro*) and in the Mediterranean regions of Africa, Europe and the Middle East (*V. orientalis*). In the 19th century, the European hornet, *V. crabro*, was intentionally released in North America to control forest caterpillar outbreaks [12], where it became established [13]. In recent years, another two species, *V. velutina* and *V. mandarinia*, were accidentally introduced respectively into Europe, Korea and Japan (*V. velutina*) [14,15,16,17,18] as well as in North America (*V. mandarinia*) [19,20]. A number of hornet species have been more or less extensively studied in their biology, either because of their social habits [2,3] or their invasive potential, with subsequent impacts on native biodiversity, on human health [21,22,23,24] and, especially, on honey bees and apiculture [25,26,27]. In fact, various hornet species have been reported to prey on different species of honey bees in Asia (Table 1). In Europe, *V. crabro* is a mild predator of *A. mellifera* [28], although its predatory pressure varies with location from year to year and seems to increase over the time (pers. obs.). Conversely, *V. orientalis* represents a serious apicultural pest in the Mediterranean region [29,30] as well as the invasive *V. velutina nigrithorax* in the invaded European countries [27,31].

In the hornet annual colony cycle, future queens emerge in spring from their winter diapause, then start alone building their new colony and occasionally visit apiaries in search of food for the few developing larvae inside their nest [2]. After the first generation brood has been reared and workers emerge, the nest size and colony population increase throughout the summer, reaching a peak in autumn, when the demand for food is the greatest to rear the new reproductive individuals (i.e., males and future queens) and bee colonies are at a greater risk of predation [2,27].

## 2. Coevolving Prey and Predator

Arms races between prey and predator lead to elaborate defensive and aggressive strategies put in place by the different actors of these antagonistic relationships [46]. Adaptation by one species generates selection on its partner to adapt and vice versa, creating an escalating arms race of counteradaptations. Under this constant selective pressure, traits and strategies evolve to maximize the chances for prey to escape the predator and for the latter to successfully catch its prey [46]. The evolutionary arms race between honey bees and predatory hornets is an ancient process, as demonstrated by the number of adaptations and counteradaptations adopted by both these social hymenopteran prey and predator [1,2,3,4,5,6,7,28,44,47,48]. The close predator–prey relationship between hornets and honey bees has been likely established over evolutionary time due to the fact that the phylogeny of both the genuses *Vespa* and *Apis* has largely taken place in the same geographic area in Asia [2,3,8,9,10,49,50]. Hornets represent major natural enemy of honey bees, as workers of different species specialize to locate and besiege honey bee colonies, hunting for foragers to feed the developing brood inside their nests [1,5,7,34,47] (Table 1). Indeed, hornet larvae need large quantities of protein food to develop, and honey bees represent the preferred prey for various hornet species when compared to other potential insect prey and non-prey protein items [51,52]. The array of mutual adaptations evolved by hornets and honey bees span from the ability of both predator and prey to detect and exploit chemicals and pheromones used by their counterpart to locate the prey or spot the predator, to the use of visual, acoustic and vibrational signals for intra- and interspecific communication, up to the hornet persistent siege or destructive mass attacks and the collectively coordinated defense response of bees. All these captivating strategies will be described in detail in the present review.

## 3. Hornet Predatory Strategies

The hornet’s ability to prey honey bees is favored by a number of morpho-physiological and behavioral adaptations. *Vespa* hornets are, in fact, well equipped for the hunting of bees: they have a larger body size compared to their prey, a heavy chitinous armor to resist bee attacks and their strong mandibles and venomous sting make them a deadly nemesis for honey bees of different species [1,3] (Table 1). As concerns the hornet’s hunting strategies, these predators must first locate their prey, and it has been experimentally demonstrated that some species, such as *V. tropica* and *V. velutina*, can use both visual and olfactory cues for the long-range detection of honey bee colonies [53,54]. Foragers of *V. tropica* can readily associate color and shape with potential food sources and exhibit color generalization [53]. *V. velutina* foragers visually distinguish between bee dummy bait and cotton ball dummy bait, both treated with bee odors, preferring bee dummies [54]. Foraging hornets are also selectively attracted to honey bee colony odors, in particular honey and pollen, as well as honey bee pheromones, which may signal a high prey density [51,53,54]. In laboratory assays, workers of *V. velutina* oriented especially towards geraniol, a component of the honey bee worker aggregation pheromone, that could therefore represent an honest signal for hornets [51]. Behavioral, chemical and electrophysiological analyses have also demonstrated that *Vespa bicolor* is attracted to (Z)-11-eicosen-1-ol, which is a major compound in the alarm pheromones of both Asian (*Apis cerana*) and European (*Apis mellifera*) honey bees, and its antennae respond to this compound [33]. Intriguingly, this hornet attraction to the specific honey bee pheromone is also exploited by the orchid *Dendrobium sinense*, which mimics the honey bee alarm pheromone in its flowers’ scent to attract hornets that visit and pollinate the flowers. It is therefore likely that the bee-hunting hornets visit the non-rewarding flowers in search of their honey bee prey [33].

**Figure 1:**
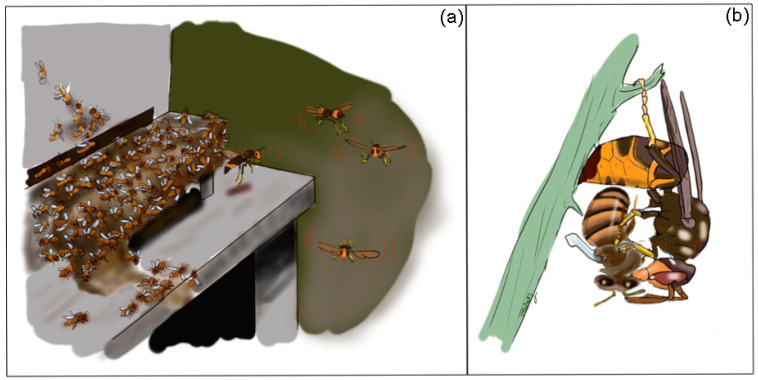
(**a**) Bee-hawking of *Vespa velutina* on a honey bee colony. Hunting hornets hover in front of a beehive trying to catch bee foragers leaving or returning to the nest after their foraging flights. (**b**) *Vespa velutina* forager manipulating its honey bee prey © J. Poidatz.

Once they have located a honey bee colony, hornets must enact effective tactics to catch their bee prey. Hornet attacks can range from solitary individuals either hunting bee foragers away from their colonies or approaching the nest entrance to catch flying foragers returning to their nests [28,40,55], up to mass slaughter and plundering of entire bee colonies [3,5,34]. A characteristic strategy adopted by different hornet species (e.g., *V. velutina*) for preying honey bee is hovering in front of their nest entrance to hawk bees from the air [55] (Figure 1a). Bee-hawking represents a low-risk strategy for the hornet compared to approaching the nest entrance to snatch a bee, because in this case, the hornet might be captured and killed by the carpet of bees defending the colony (see below). During hawking, a hornet takes up a specific posture: it hovers in front of a bee colony, facing away from the entrance with its legs stretched backwards to hunt returning foragers [44,55].

A hornet can hover nearby a honey bee colony for more than 25 min in the attempt to catch a bee and will also approach the nest entrance, landing near the defending bees to try and snatch a prey [55]. After catching a bee, the hornet usually flies to a support where it manipulates its prey (Figure 1b). The predator usually starts by beheading the bee and then with its mandibles cuts off the abdomen and crushes the bee thorax before taking the dismembered prey back to the nest to feed the larvae that require protein food to develop [55]. The persistent attacks progressively weaken honey bee colonies, often leading to their death due to the intense predation pressure or to the inability to collect a sufficient amount of resources to survive the overwintering period, with potential colony losses of up to 30% [36,55,56,57,58,59]. Indeed, the most intense period of hornet hawking over apiaries and feral colonies usually coincides with floral dearth periods, in late summer or early autumn at temperate and subtropical latitudes [28,60]. Then the hornet nests are at their peak in terms of immature brood population (i.e., larvae) in need of feeding with protein resources, while nectar and pollen sources for the honey bees are scant and insufficient [2,60].

**Figure 2:**
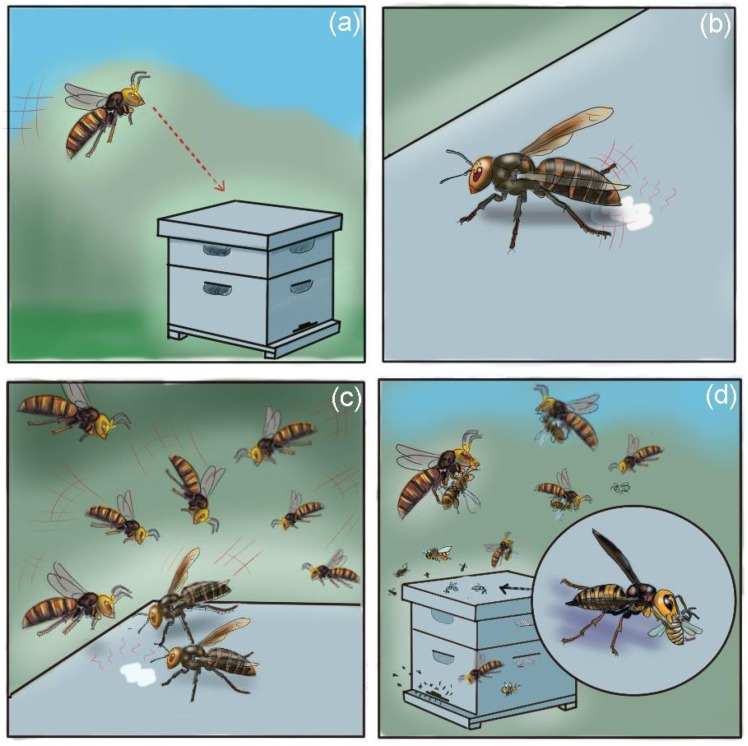
*Vespa mandarinia* predatory strategy on honey bees: (**a**) hornet scout spots a bee colony, (**b**) pheromone-marking phase, (**c**) recruitment phase and (**d**) slaughter/occupation phase. © J. Poidatz.

In the most extreme cases, as for example with the giant hornet *V. mandarinia*, which has evolved a group predation strategy against other social bees and wasps [2], hornet workers can carry out destructive raids on honey bee nests, both feral and managed, exterminating entire colonies and ravaging their brood and resources within a few hours [1,2,5,34]. These attacks en masse usually start with a single hornet scout locating a bee colony (Figure 2a); then, the scout marks the prey’s nest with a pheromone, 1-methylbutyl 3-methylbutanoate [61], from its van der Vecht and venom gland, which recruits nearby nestmates [2,5,61,62] (Figure 2b,c). Once several dozens of nestmate hornets have gathered around the bee colony, the “slaughter phase” [2] (Figure 2d) begins with hornets approaching the nest entrance, killing thousands of bees trying to defend their nest. The bees’ defenses are quickly overwhelmed by the attacking hornets and the colony is killed or it absconds, leaving behind a large quantity of brood and resources, which are plundered by hornets for several days after the attack [5,34,36,48].

## 4. Honey Bee Defense Strategies

**Figure 3:**
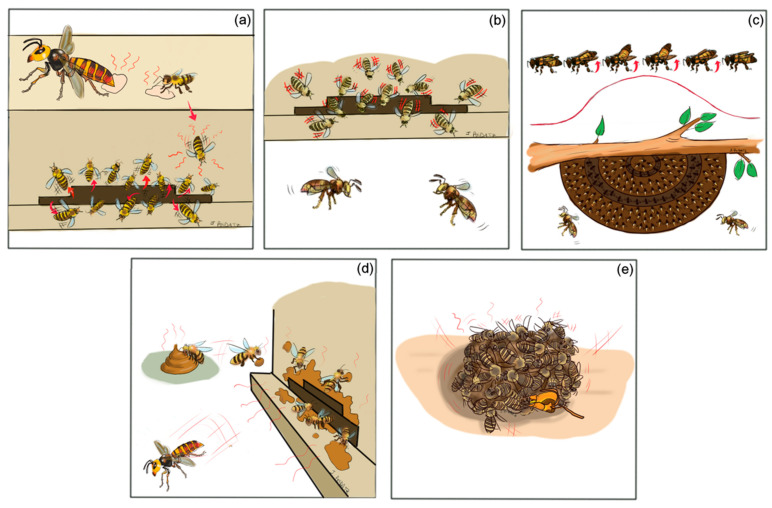
Honey bee defense strategies against hornets: (**a**) pheromone detection, (**b**) shimmering display in *A. cerana*, (**c**) Mexican wave-like shaking in *A. dorsata*, (**d**) filth-spotting at the hive entrance and (**e**) heat-balling. © J. Poidatz.

Despite the intense siege of bee-hawking hornets and the utter brutality of these raids, honey bees are not left without defenses. The honey bees, especially the Asian ones, which have evolved in sympatry with various hornet species, appear to have developed a number of defensive collective strategies to effectively reduce predatory pressure and prevent these destructive attacks [63]. These collective strategies can be divided into those first lines of defense adopted by bees to quickly detect the hornet presence and repel its approach, and the more active reactions that bring the defenders into physical contact with the hornet menace in the attempt to eliminate it. As regards the first line of coordinated group defenses, for example, workers of the Asian honey bee, *A. cerana*, have evolved the ability to detect the marking pheromone used by hornet scouts to recruit nestmates and respond to it by increasing the number of defenders at the nest entrance, trying to lure the lone scent-marking hornet scout to attack it in group before its pheromone attracts other hornets [5,48] (Figure 3a). Such an olfactory eavesdropping in Asian honey bees has also been observed towards the alarm pheromones, such as 1-methylbutyl 3-methylbutanoate (1-M-3-MB), 2-pentanol (2-P) and 3-methyl-1-butanol (3-M-1-B), emitted by different hornet species [48,64]. *A. cerana* workers respond to these components in the alarm pheromone of *V. mandarinia* by exiting their hive and increasing their activity at the nest entrance [48] and form heat-balls (see below) [4,5] around hornet lures in response to a synthetic blend of the *V. velutina* alarm pheromone [64]. Furthermore, electroantennography (EAG) demonstrated that the antennae of *A. cerana* workers show a strong and consistent response to *V. velutina* alarm pheromone compounds [64]. EAG and conditioning experiments demonstrated that *A. cerana* bees can also detect the odor of live hornets, and this detection affects their olfactory learning and foraging efficiency by reducing the bee foragers’ ability to learn floral odors [65]. The defensive behavior of Asian honey bees towards predatory hornets is not only triggered by chemical cues. When the visual stimulus of a hornet, especially *V. velutina* and *V. simillima*, is presented to a colony of *A. cerana*, the guard bees at the nest entrance recruit a high number of nestmates [55] and perform a synchronized “shimmering” [6,49,66,67,68] (Figure 3b). Such a shimmering display consists of a coordinated rapid shaking of the bees’ abdomens that is repeated at approximately one-second intervals and is accompanied by a loud buzzing noise [6,68]. The production of hissing or buzzing sounds as aposematic signals in response to hornets and other predators has been observed in various species of honey bees [69,70,71].

The shimmering display, which appears to be innate in *A. cerana* [68], with its visual, chemical, acoustic and vibratory components, represents a sort of prey–predator “I see you” signal that, apart from alerting incoming nestmate foragers of the predator presence, should also inform the hornet that it has been spotted by the colony, which is ready to defend against it [6,72]. As hornets get closer, a higher number of bees are recruited, and the shimmering strength increases, indicating that *A. cerana* could evaluate the predation-risk level, and the intensity of the display is finely tuned to the movement and distance of the predator [6]. The primary function of shimmering to deter hornets is confirmed by the fact that foragers of *V. velutina* respond to the display by not approaching the colony, and the bee predation rate is consequently reduced [6,72]. Through the shimmering display, honey bee colonies can therefore enact a first line of defense against hornets without physical contact with their larger and heavily armored enemies, thereby minimizing the risk for the defending bees. When bee colonies are besieged by hunting hornets, the shimmering signal can also alert individual bee foragers to alter their flight styles in response to avoid being captured by hawking hornets [55]. Such alterations in the flight pattern in response to the hornet presence has been documented for *A. cerana*, whose foragers increase their flying speed up to 20 times and fly in straight for the nest entrance covered by the carpet of shimmering bees [55]. The menace of a hornet attack can also reduce foraging activities in *A. cerana* colonies through the production of a vibrational “stop signal” emitted by workers, which inhibits waggle dancing and forager departure from the colony [7,73,74]. Once more, the intensity of the signal is tuned to the severity of the threat, since the larger and more dangerous *V. mandarinia* elicits a higher vibrational amplitude in the signal, which results in a more effective inhibition of foraging activities, with respect to the smaller *V. velutina* [7,73,74].

Although documented in Asian cavity-dwelling bees (*A. cerana* and *A. nuluensis*) [6,40,68], the shimmering behavior against predatory hornets has probably evolved first in those species of bees nesting in the open, as it has also been documented in the dwarf honey bees, *Apis florea* [75,76], and in the giant honey bee, *A. dorsata* [38,77,78,79]. In the latter, the shimmering display represents one of the most striking lines of defense against potential predators as bees covering the colony comb produce highly coordinated Mexican wave-like cascades, whereby hundreds of workers flip their abdomens upwards to repel the approaching enemy [49,66,69,77,78,79,80,81,82] (Figure 3c). The wave-like shimmering over the *A. dorsata* comb is triggered mainly by visual stimuli, especially in the case of hornets hovering in near proximity of the colony [38,77,82]. The collective body shaking, generated at distinct spots of the comb surface [79,82], spreads over the whole colony within a fraction of seconds, producing the characteristic Mexican wave-like pattern [79,82,83,84]. As for *A. cerana*, the shimmering display can instantly bring hundreds of nestmate bees to flip their abdomens [6,79,82,83,84]. This rapid recruitment transmits information to both colony members, stimulating them to participate, possibly alerting them through mechanoceptive and pheromonal communication [84], and to potential predators, by providing dynamic visual cues that might confuse or repel approaching enemies [77,84]. These wave-like displays are evoked primarily by hornets [38,77] and become stronger and more frequent the nearer to and the faster the hornet approaches the colony, while they seem quite ineffective in deterring larger predators such as birds or mammals [85].

Another interesting behavior by *A. cerana*, which can be counted among the collective defenses to prevent hornet attack, and was only recently documented in Vietnam by Mattila et al. [41], consists of bee workers foraging for filth (e.g., animal feces) and applying it in mounded spots around their nest entrances in response to hornets (Figure 3d). Such a curious spotting strategy is initiated specifically when bee colonies are exposed to natural attacks from *Vespa soror* and to its nest-marking pheromones [41]. The study has demonstrated that moderate to heavy spotting on a colony reduces the likelihood and the time *V. soror* foragers spend attempting to breach the nest entrance by chewing on it and also lowers the percentage of attacks involving multiple hornets [41]. Interestingly, *A. cerana* workers in the same geographic area do not apply feces over nest entrances when they are attacked by other hornet species, such as *V. velutina*. Such difference likely reflects the diverse hornets’ hunting tactics, that is, mass attacks with entry and destruction of the bee nest for *V. soror*, against hawking of individual bees for *V. velutina* [55] and, therefore, the different level of threat that they each pose to colonies [41]. The intriguing results of this work constitute the first report of honey bees using animal droppings to deter attacks by a specific hornet predator.

When the first lines of defense fail in keeping away hornets from their colonies, honey bees must respond to the predator attack by trying to catch and kill those hornets that come too close to the carpet of defending bees. One of the most characteristic and deadly defense behaviors developed by honey bees against predatory hornets is heat balling of hornets that come into direct contact with the honey bee nest. This coordinated defensive behavior has been reported for *A. cerana*, *A. mellifera*, *A. florea* and *A. dorsata* [4,5,28,47,59,69,86], but it has been extensively studied in *A. cerana*. The approach of a hornet to a nest or the detection of the marking pheromone emitted by hornet scouts cause a massive recruitment of *A. cerana* workers, which gather at the nest entrance, apparently to draw the hornet further into the colony. If the hornet enters the nest or gets in touch with the bees, they immediately seize the intruder in a tight ball, then use their flight muscles to generate heat, decrease oxygen income and increase the CO_2_ level, which kills the hornet [4,5,59] (Figure 3e). The number of bees recruited to the ball depends on the hornet species, ranging from less than 100 workers surrounding a forager of *V. velutina* [87], to 180–300 workers against the solitary hunting *V. simillima xanthoptera* [4], up to more than 400–500 engulfing the larger predator in the case of the giant hornet *V. mandarinia* [5]. Regardless of the number of bees involved, the defensive strategy proves highly effective as hornets are killed in about 10–20 min after being surrounded by the living ball [4,5,87,88]. The increasing number of bees according to the different threat and the compactness of the ball assures that the temperature inside the ball is raised sufficiently high to kill the captured hornet at its core but not the bees [4,5,59,87,88]. Indeed, the lethal temperature limit for the bees is 48–52 °C, while the different hornet species have a thermal tolerance between 44–47 °C [5,59]. Ono et al. [5] reported that *A. cerana* workers do not sting the captured hornet, although very recent research has demonstrated that bee stings also increase hornet mortality alongside heat [89]. Ono et al. [5] also suggested that isoamyl acetate, a component of the honey bee alarm pheromone, is emitted by bees forming the ball, and its evaporation, aided by the increased temperature inside the ball, may act as a recruitment signal for other bees to join.

In more recent years, however, it has been demonstrated that the trapped hornet is not merely baked to death by the heat developed by the large mass of vibrating bees [86]. In fact, giant hornets *V. mandarinia* are often killed in less than 10 min when they are engulfed in a bee ball, but, in controlled experiments, the same hornets are able to survive for 10 min at the temperature up to 47 °C, whereas the temperature inside the bee balls does not exceeds 46 °C [88,90]. Sugahara and Sakamoto [88] have demonstrated that hornets at the center of the heat ball are also asphyxiated due to the carbon dioxide generated by the bees. They have discovered that the CO_2_ concentration inside the bee ball reaches a maximum of 3.6 ± 0.2% within the first 5 min after the bee ball formation and the lethal temperature of the hornet under conditions of CO_2_ concentration (3.7 ± 0.44%) corresponds to 45–46 °C, which is comparable to the one measured inside the bee ball [88]. Thus, it appears that the high level of CO_2_ produced by honey bees inside the bee ball plays a major role alongside temperature in the effective defense against hornets.

## 5. Less Effective Defense of *A. mellifera* against Hornets

Differently from the Asian species of the genus *Apis*, the Western honey bee, *A. mellifera*, has differentiated in Africa, the Near and Middle East and Europe [91]. In these geographic areas, only two species of hornets are naturally present, *V. crabro* and *V. orientalis*, and, although both are honey bee predators [28,44,52,71], the selective pressure they exert on their prey is undoubtedly lower when compared to the persistent and lethal attacks carried out on bee colonies by Asian hornets such as *V. velutina*, *V. simillima*, *V. mandarinia* or *V. soror* [3,4,5,41,92]. This relative lack of coevolution between *A. mellifera* and more dangerous *Vespa* hornets has resulted in less efficient and organized collective defenses of Western honey bees against hornets [28,55,93]. This less effective defense abilities put the survival of *A. mellifera* at risk when it comes into contact with these deadly predators, similar to Western honey bee colonies that were first imported in the early 20th century to Asia for commercial purposes [59,94,95], or in the more recent cases of invasive Asian hornets accidentally introduced in Europe and North America [15,19].

Similar to Asian honey bee species, *A. mellifera* also shows shaking displays and balling behavior to some extent, but both defense strategies achieve a lower efficacy against hornets [28,55,93,96,97]. The defensive reaction of *A. cerana* is, in fact, more efficient in terms of both the number of recruited workers and the increase in balling temperature [30,55,59,73,96]. In *A. cerana*, the average number of guard bees recruited in the presence of hawking hornets increases eightfold per minute, from around 3 to almost 27 guards, while in *A. mellifera,* the number simply increases from around 6 to less than 10 guards recruited per minute [55]. Moreover, when workers of the two species perform the heat-balling behavior towards hornets, in *A. cerana,* up to more than 500 bees quickly engulf the hornet, reaching a ball temperature of 46–47 °C [4,5], which is sufficient to kill different species of hornets [5,59], whereas the temperature reached inside the less crowded and coordinated ball formed by *A. mellifera* workers does not exceed around 44 °C [28,30,96], which is below the thermal limit of various hornet species. However, Western honey bees are still able to perform effective defensive mechanisms against hornets as suggested by the behavioral response against hornets in several *A. mellifera* subspecies and the discovery of dead hornets in front of beehives [28,30,45,98,99].

The subspecies *A. mellifera cypria*, for example, shows peculiar adaptations against its hornet predator *V. orientalis* [98]. The subspecies adopts contrasted defensive tactics at the colony level, with some colonies rapidly increasing the number of guards at the hive entrance to attack, engulf and kill approaching hornets, while others avoid direct conflicts with the predator by gradually retreating to form a defensive line of honey bees at the hive entrance [98]. The tendency of some colonies to retreat appears to be positively correlated with the deposition of propolis walls with small apertures at the hive entrances to prevent the hornets’ entry into the colony [98]. Cyprian honey bees also produce a characteristic hissing sound of high frequency, which might represent an alarm signal towards conspecifics and an aposematic signal for hornets [71]. *A. mellifera cypria* also adopts a balling defense against *V. orientalis* [30], but the strategy is not equivalent to the thermo-balling reported for *A. cerana*, since the temperature inside the ball does not reach the hornet thermal limit and bees kill the predator by blocking its respiration instead [30]. In fact, the reached temperature in the core is 44 ± 0.5 °C, while *V. orientalis* is able to tolerate a temperature up to 50.6 ± 0.6 °C [30].

The effective anti-hornet response of *A. mellifera cypria* is probably due to its sympatry with *V. orientalis* and the subsequent high predation risk that this species has to face [30,98]. Indeed, other subspecies, such as *A. mellifera caucasica* and *A. mellifera meda,* exhibit a much less effective defense against *V. crabro* and *V. orientalis* [45,99]. Additionally, *A. mellifera ligustica* shows some degree of defense against its sympatric predator *V. crabro* [28]. The European hornet predatory strategy consists in patrolling hives’ entrances to spot and then swoop on returning bee foragers [28]. In this case, honey bee defense is centered on packed aggregations of guards at the hive opening to deter approaching hornets from landing. Moreover, bees coordinately cling together in groups to catch the hornet and then cover it in a typical balling behavior [28]. It is likely that a combination of over-heating alongside carbon dioxide emission and venom release by the balling bees may contribute to the hornet death, since, as for *A. mellifera cypria* against *V. orientalis* [30], the mere increase in temperature inside the ball does not always reach the hornet lethal limit [28]. Indeed, while the lethal temperature measured for *V. crabro* was 44.2 ± 0.5 °C, the maximum temperature recorded at the core of the bee balls was 39.9 ± 7.4 °C, and almost 60% of tested colonies could not reach temperatures higher than 43 °C. Overall, the defense behavior of *A. mellifera ligustica* appears adequate to partially counteract at least the European hornet *V. crabro*, probably due to a long co-adaptation in a temperate environment, while it seems ineffective against other predators such as *V. orientalis*, *V. velutina* or *V. mandarinia* [28]. In fact, different from Asian honey bees, *A. mellifera* colonies introduced in Asia do not perform the shimmering signal towards hornet predators [6], do not respond to the marking pheromone used by *V. mandarinia* scouts in the preliminary phase of the group attacks [48] and do not reduce their foraging activity in the presence of *V. velutina* hornets [55,59]. Bee foragers returning to the hive are instead disoriented by hawking *V. velutina* hornets and slow down their flight speed, thus increasing the exposure time to the predator [55]. As a result, *V. velutina* hawking success rates in terms of captured prey are about three times higher for *A. mellifera* than for *A. cerana* [55].

## 6. Invasive Threats

The inability of Western honey bees to efficiently defend themselves against allopatric *Vespa* species poses a serious issue in the case of invasive alien hornets [100]. The Asian yellow-legged hornet, *V. velutina nigrithorax*, in particular, has represented an emerging problem for European honey bees and commercial beekeeping since its first record in France in 2004 [15]. The species, which has also successfully invaded Korea and Japan [14,17], has been spreading across most of Western Europe, including France, Italy, Spain, Portugal, Belgium, Netherlands, United Kingdom, Germany and Switzerland [15,27,101,102,103,104,105,106,107,108,109,110]. The rapid spread of *V. velutina* in Europe has significant ecological, economic and public health impacts, leading to its inclusion in the List of Invasive Alien Species of Union concern [27,31,111]. Apart from the risks for human health due to the species habit to nest in urban environments, which increases the likelihood of venomous stings for humans [22,23,112,113], the yellow-legged hornet preys on a vast array of insect species [114,115] and it is likely to compete with native species that share a similar ecological niche [52,116]. However, the main threat posed by *V. velutina* is on beekeeping activities, due to its intense predation carried out on honey bees and their lack of effective defense strategies [27]. Indeed, the yellow-legged hornet heavily hunts honey bees of the genus *Apis* in both native and invaded areas [55,59,60,92,93,116,117], and honey bees accounts for a considerable part of dietary protein in the hornet diet [118,119].

In the European countries invaded by *V. velutina*, *A. mellifera* colonies are facing its impact year after year, with reported losses of at least 20–30% [120,121]. Nonetheless, despite the intense predation pressure of the invasive *V. velutina* on European apiaries, *A. mellifera* exhibit a largely inefficient and unorganized defense against this hornet, unlike honey bees from other areas around the globe, where they have co-evolved in sympatry with their natural *Vespa* predators [27,31,92,93,97].

The giant hornet, *V. mandarinia*, could pose another significant invasive menace with a heavy impact for Western honey bees, since the species has recently been spotted in the USA, in the state of Washington [10,20,122]. Models based on climatic suitability in North America suggest that, without adequate control, the species could successfully establish populations across North America and the predicted suitable areas largely overlap with areas where honey production is highest, as well as with species-rich areas for native bees [20,122,123]. Thus, *V. mandarinia* may have major economic and environmental impacts, and it appears urgent and necessary to control this starting invasion [20,122].

Invasive hornets could also represent a threat to honey bees in the form of potential vectors of pathogens and diseases. Recent research has in fact demonstrated that honey bee viruses can be found in both native and invasive hornet populations [124,125,126,127,128]. Since viruses can often move from primary hosts to new hosts and a number of bee viruses have been identified in hornets [125,126,127,128], infected hornet predators might play a role in the host dynamics of honey bee viruses [127]. Foraging hornets can hunt for bees across broad areas; therefore, they could come in contact with, carry and potentially transmit viruses from multiple honey bee colonies [127].

## 7. Coevolution as a Tool to Develop Sustainable Management Strategies

The evolutionary arms race between hornets and honey bees provides not only fascinating examples of adaptations and counteradaptations between predator and prey, but might also represent a starting point for the development of effective and sustainable strategies to protect honey bees and beekeeping activities and to control invasive alien species of hornets. Indeed, deepening the knowledge on the biology of these species and the interaction with their prey appears crucial to successfully face their threat.

The knowledge of the foraging strategies and predation dynamics of different hornet species, for example, can be exploited to deploy traps in field when the predatory pressure over apiaries and beehives is at its peak to contain the number of hunting hornets. For the invasive *V. velutina* in France, it has been observed in the field that, at this latitude, predation lasts for more than 5 months and can be divided into three main phases [89]. The first one, from the beginning of the season till early summer, is when very few hunting hornets are present in apiaries. This is followed by a large increase in predation on honey bee colonies from early August to early November, due to the hornets’ need of protein sources to rear the new generation of sexuals inside their nest. Finally, predation drastically decreases from November to December, after the emergence of reproductive individuals, when the colony reaches the end of its cycle [2,27]. Moreover, by analyzing the trapped hornet and measuring different parameters, such as individual body mass variation or fat storage, it is possible to infer the hornet population dynamics [92,129,130], which can be useful to adopt effective management strategies. In fact, hornet foundresses can be captured in traps in early spring, while foraging for sugary solutions or prey to feed their first brood in the nest, whereas reproductive gynes can be trapped in late autumn before they enter hibernation, and the trapping of reproductive females before or after hibernation can reduce the establishment and the survival of new colonies [27].

The hornet dietary preferences and specific attraction towards honey bees and their pheromones [51,52,53,54] could also be exploited to create selective baits to control hornets’ populations and their predatory pressure. Toxic-baiting strategies using non-selective baits and nest destruction have proven to be effective management strategies against some invasive hornets and wasps in different geographic areas [131,132]. Nonetheless, the use of non-selective baits and traps is not environmentally sustainable due to their absence of species specificity and the consequent threat they pose to numerous species of the native entomofauna [27,132,133,134]. Experiments carried out both in the laboratory [51,52] and in the field [53,54] have demonstrated that different species of hornets prefer honey bees with respect to other protein sources [52] and are attracted to honey bee products and odors [51,53,54]. These hive products, such as honey, pollen and propolis, or pheromones, such as geraniol (honey bee aggregation pheromone), β-ocimene (brood pheromone), homovanillyl alcohol and methyl-4-hydroxybenzoate (queen pheromones) [51], could therefore be used as selective attractants for hornet trapping alongside specific hornet pheromones [27,51,52,53,54,134,135,136]. However, the use of honey bee odors and pheromones as selective bait may pose some problems in apiaries, since these specific compounds could also attract honey bees and disturb honey bee colonies and their activity. To solve this potential issue, other specific volatiles produced by different sources have been and might further be tested [137]. For example, it has been demonstrated that a specific strain of bacteria (*Bacillus* sp. BV-1) [137] produces volatile compounds that attract *V. velutina* foragers. These compounds, such as the 3-methyl-1-butanol, which has been reported as a likely attractant [137], could be used in the field for a sustainable management of invasive hornets.

The defensive strategies adopted by various species or lineages of honey bees and their different anti-predatory effectiveness can also provide a basis to develop a sustainable management towards invasive alien hornets. In fact, as described above, *A. cerana* or *A. mellifera cypria* are able to successfully defend against the species of hornets they have coevolved with, and this defensive ability could be exploited in the field to protect apiaries through the selection of bee lineages capable of repelling hornet attacks [87,98,99].

## 8. Conclusions

Investigating the coevolutionary adaptations between honey bees and their hornet natural enemies grows increasingly timely as anthropogenic changes in the environment are quickly happening, with a progressive decline of honey bee populations [138]. Among the causes of such decline, it is possible to identify the massification of beekeeping activities and the increased global trade, which favors the introduction of new threats for honey bees [138]. In recent decades, invasive hornet species and their impact have been added up as potentially severe threats to bees and beekeeping in different regions [15,16,20,22,27,123]. Despite that, sustainable management techniques to keep invasive hornet menaces at bay and to protect both feral and managed honey bees still need to be improved [132,139]. In this regard, the study of the complex relationship between honey bees and hornets provides not only a large array of finely tuned adaptations and counteradaptations by predator and prey, which are relevant in a natural history perspective, but also a possible basis to develop effective and sustainable hornet control techniques. In this context, it is crucial to review and expand the knowledge upon the many examples of antagonistic interactions between honey bees and hornets so that we can understand and appreciate the importance of this coevolutionary arms race and its potential implications for honey bees’ protection.

## Figures and Tables

**Table 1:** Hornet species of the genus *Vespa* preying on *Apis* spp. in different geographic areas.

Geographic Area	Native Hornet Predator	Introduced Hornet Predator	Native Honey Bee Prey	Introduced Honey Bee Prey	References
China	*Vespa velutina*, *V. bicolor*, *V. mandarinia*, *V. tropica*	/	*Apis cerana*	*A. mellifera*	[6,7,32,33]
Japan	*V. mandarinia*, *V. simillima*	*V. velutina*	*A. cerana*	*A. mellifera*	[4,5,17,34]
India	*V. velutina*, *V. magnifica*, *V. orientalis*, *V. basalis*, *V. tropica*, *V. affinis*	/	*A. cerana, A. dorsata*	*A. mellifera*	[35,36,37,38]
Thailand	*V. tropica*	/	*A. cerana*	*A. mellifera*	[39]
Borneo	*V. multimaculata*	/	*A. nuluensis*	*A. mellifera*	[40]
Vietnam	*V. soror*	/	*A. cerana*	*A. mellifera*	[41]
Korea	*V. analis*, *V. crabro, V. dybowskii*, *V. mandarinia*, *V. simillima*	*V. velutina*	*A. cerana*	*A. mellifera*	[14,42,43]
Middle East	*V. orientalis*, *V. crabro*	/	*A. mellifera*	*/*	[44,45]
Europe	*V. orientalis*, *V. crabro*	*V. velutina*	*A. mellifera*	/	[15,16,27,28,29,30,31]
North America	/	*V. crabro*, *V. mandarinia*	/	*A. mellifera*	[12,13,19,20]

## Data Availability

Not applicable.
